# Potential Effect of Glutamine in the Improvement of Intestinal Stem Cell Proliferation and the Alleviation of Burn-Induced Intestinal Injury via Activating YAP: A Preliminary Study

**DOI:** 10.3390/nu15071766

**Published:** 2023-04-04

**Authors:** Xia Chen, Panyang Zhang, Yajuan Zhang, Shijun Fan, Yan Wei, Zhifan Yang, Fengchao Wang, Xi Peng

**Affiliations:** 1 Clinical Medical Research Center, Southwest Hospital, Third Military Medical University (Army Medical University), Chongqing 400038, China; chenxia05201995@163.com (X.C.); zhangpanyang1@126.com (P.Z.); zyj18696625642@126.com (Y.Z.); fanshijun1211@hotmail.com (S.F.); weiyan56333849@163.com (Y.W.); 2 Institute of Combined Injury, State Key Laboratory of Trauma, Burns and Combined Injury, College of Preventive Medicine, Third Military Medical University (Army Medical University), Chongqing 400038, China; yang1269043@163.com; 3 State Key Laboratory of Trauma, Burns and Combined Injury, Third Military Medical University (Army Medical University), Chongqing 400038, China

**Keywords:** glutamine, intestinal stem cells, crypt, proliferation, burns

## Abstract

Burn injury is a common form of traumatic injury that leads to high mortality worldwide. A severe burn injury usually induces gut barrier dysfunction, partially resulting from the impairment in the proliferation and self-renewal of intestinal stem cells (ISCs) post burns. As a main energy substance of small intestinal enterocytes, glutamine (Gln) is important for intestinal cell viability and growth, while its roles in ISCs-induced regeneration after burns are still unclear. To demonstrate the potential effects of Gln in improving ISCs proliferation and alleviating burn-induced intestinal injury, in this study, we verified that Gln significantly alleviated small intestine injury in burned mice model. It showed that Gln could significantly decrease the ferroptosis of crypt cells in the ileum, promote the proliferation of ISCs, and repair the crypt. These effects of Gln were also confirmed in the mouse small intestine organoids model. Further research found that Yes-associated protein (YAP) is suppressed after burn injury, and Gln could improve cell proliferation and accelerate the renewal of the damaged intestinal mucosal barrier after burns by activating YAP. YAP is closely associated with the changes in intestinal stem cell proliferation after burn injury and could be served as a potential target for severe burns.

## 1. Introduction

Intestinal injury is a common complication in severe burn patients. The damaged intestine not only affects the absorption of nutrients, leading to malnutrition in patients, but more importantly, it is the pathophysiological basis of gut-derived infection and enterogenic hypermetabolism, which is an important factor leading to poor prognosis [1,2]. Various studies have shown that the repair and functional re-establishment of the damaged intestinal mucosal barrier relies on a reserve stem cell population; its proliferation and self-renewal are key factors in intestinal tract injury repair [3,4]. When the intestine is irritated by external damage, the repair function of the stem cells is initiated through relevant mechanisms at the molecular level, which stimulates the intestinal stem cell (ISC) proliferation and generates progeny cells to substitute the damaged intestinal epithelial cells and regenerate the intestinal barrier [5]. During the process of stem cell division and proliferation, their demand for nucleic acid and protein increases due to DNA replication and protein synthesis [6,7]. Therefore, the proliferation of ISCs is highly dependent on anabolism. In this process, in addition to the essential growth stimulation hormones and growth factors, some special nutrients, including glutamine (Gln), glucose, ascorbic acid, and lipids can promote cells anabolism, improve the proliferation activity of stem cells, and accelerate the repair of damaged intestinal mucosa [8]. Among these nutrients, the role of Gln is particularly noteworthy.

Gln is the most plentiful amino acid in mammals, accounting for more than half of the free amino acids [9]. In recent years, Gln has aroused extensive concern in view of its significant metabolic changes in diseases; several studies have shown that Gln is an important precursor for the synthesis of pyrimidine, purine nucleotide, and amino sugar as a conditionally required amino acid [10]. Gln is a precursor for the synthesis of reduced glutathione (GSH), an important antioxidant in vivo. It can improve the antioxidant capacity of the body by maintaining and increasing the GSH reserve in tissue cells [11]. Gln has been found to have trophic effects on ISCs and crypt cells. Supplementation of Gln decreased crypt depth and increased the villus/crypt ratio in three-week-old weaned mice [12,13]. These results support that Gln can promote the proliferation of ISCs, increase the formation of crypt organoids, and maintain their stability. Collectively, these studies demonstrate that Gln can promote the proliferation of ISCs, but its mechanism, especially the signaling mechanism, is not fully understood so far.

Stem cell proliferation is a complex physiological process regulated by multiple signals, in which the Hippo pathway is gradually gaining attention [14]. Recent studies demonstrated that the Hippo pathway was related to the physiological processes of stem cell proliferation, differentiation, and self-renewal [15]. The Hippo pathway is composed of several conserved kinases, such as mammalian sterile 20-like kinase 1 (MST1) and Yes-associated protein (YAP); as transcriptional coactivators, they are the main effectors of the Hippo pathway, which can promote the proliferation of ISCs and accelerate the repair of damaged intestinal epithelium [16]. Studies have reported that MST1 transcription and YAP activity are closely related to Hippo signaling pathway activation [16]. This signaling pathway is involved in the regulation of ISCs and plays an indispensable role in cell proliferation, apoptosis, differentiation, and development [17]. In a previous study, YAP could lead to the accumulation of intermediate products in glycolysis by promoting the expression of glucose transporter [18]. Studies have reported that Gln not only has glucose replacement effects but also could regulate glycolysis [19]. Therefore, we hypothesized that Gln might regulate YAP activity through the glycolytic pathway, thereby promoting the proliferation of ISCs. In this study, the expected research objectives are to find the cause of YAP changes and functional degeneration of ISCs after burns and how Gln regulates the activation of YAP to promote the proliferation of ISCs and improve the repair of the damaged intestinal mucosa.

## 2. Results

### 2.1. Gln Improves Crypt Survival and Mitigates Pathological Damage of Small Intestine after Burns in Mice

**Figure 1:**
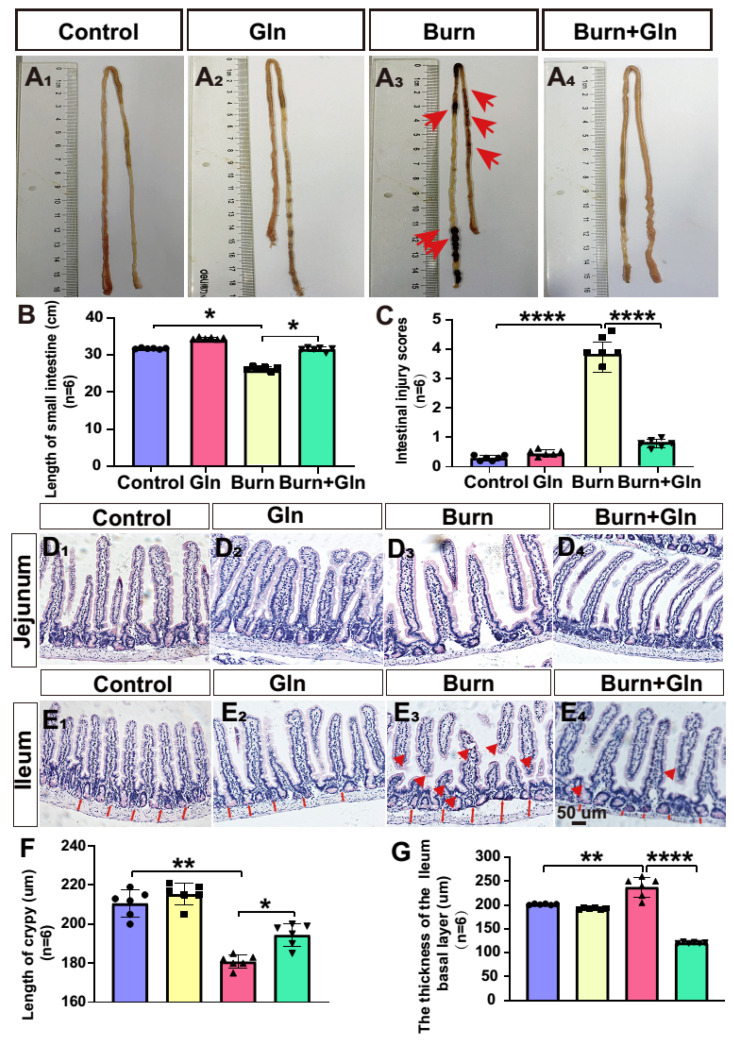
Effect of burn injury on the pathological structure of small intestinal crypts. The villus length and crypt depth were measured as indicated in the image (×100; *n* = 6). Yellow arrows indicate goblet cell infiltration in the small intestine. The red bidirectional arrows indicate the thickness of the basal tissue of the small intestinal crypt. (**A_1_**–**A_4_**) Representative images of the small intestine of each group under a light microscope: (**A_1_**) Control group; (**A_2_**) Burn group; (**A_3_**) Gln group; and (**A_4_**) Burn+Gln group. (**B**) Quantitative analysis of the length of the small intestine under different treatments. (**C**) Quantitative analysis of the number of preservation injury scores of the small intestine of different groups. (**D_1_**–**D_4_**) Representative images of the hematoxylin and eosin (H&E) staining of the small intestine sections in jejunum from control\Gln\Burn and Burn+Gln group at day 3. (**E_1_**–**E_4_**) Representative images of H&E staining of the small intestine sections in ileum from control\Gln\Burn and Burn+Gln group at day 3. (**F**) The statistical analysis of the length of crypts from images shown in (**E_1_**–**E_4_**). (**G**) The statistical analysis of the thickness of the lleum basal layer in ileum from images shown at (**E_1_**–**E_4_**). The data are Mean ± SEM with an *n* = 6. * *p* < 0.1, ** *p* < 0.01, **** *p* < 0.0001 using one-way ANOVA and post hoc Tukey’s test.

Gln is a crucial metabolic source for small intestinal cells and is important for the growth of intestinal cells. In view of the importance of Gln in intestinal homeostasis, we added Gln to observe whether it could reduce the damage of ISCs after burns. We first constructed a burned mouse model, as shown in Figure S1A; the scabby area on the back of mice was increased time-dependently. Subsequently, we found that the body weight of mice decreased significantly over time (Figure S1B). We observed the effects of Gln treatment on the small intestine under a light microscope after burns. As presented in Figure 1A–C, the intestinal tract was obviously atrophic, the length of the small intestine was significantly shortened, the intestine was perforated, and the blood vessels on the surface of the intestinal tract were increased in the Burn group. Histopathological scores for small intestine injury were used to evaluate the extent of intestinal injury (Figure 1C). Compared with the Burns group, the injury scores of small intestinal in the Burn+Gln group had significantly decreased. Next, the results of hematoxylin–eosin (H&E) staining showed that burns stress induced remarkable morphological alterations in the small intestine (Figure 1D,E). In both jejunum and ileum, the villi were disordered, the depth of crypts became shallow, and obvious vacuoles appeared in the crypts, accompanied by the thickening of intestinal basal tissues (Figure 1D–G). However, the addition of Gln could rescue the pathological damage of the crypts of small intestinal by burns. For instance, as shown in Figure 1D–F, it was found that the length of crypts in the jejunum after burns was significantly reduced compared with the control group. After Gln treatment, the length of crypts in the jejunum increased compared with the Burn group. As well, in Figure 1G, the ileum basal tissue increased significantly compared with the control group, while the basal tissue thickness decreased significantly after Gln treatment.

In addition, the results of Periodic acid Schiff (PAS) staining showed obviously visible goblet cell hyperplasia in the Burn group compared to the control group. Goblet cell expression in the Burn group was significantly higher than that in the control group (Figure S2A,B). However, we found that goblet cell proliferation was reduced after Gln treatment (Figure S2A,B). These results added further evidence for the pathological changes in the small intestine, and the damage to crypts was serious after burns. On the other hand, Gln treatment mitigated the pathological injury and improved the survival of the crypts of the small intestine, indicating its protective effects on burns-induced intestinal damage.

### 2.2. Gln Promotes Budding in Three-Dimensional (3D) Cultured Intestinal Organoids after Burns

**Figure 2:**
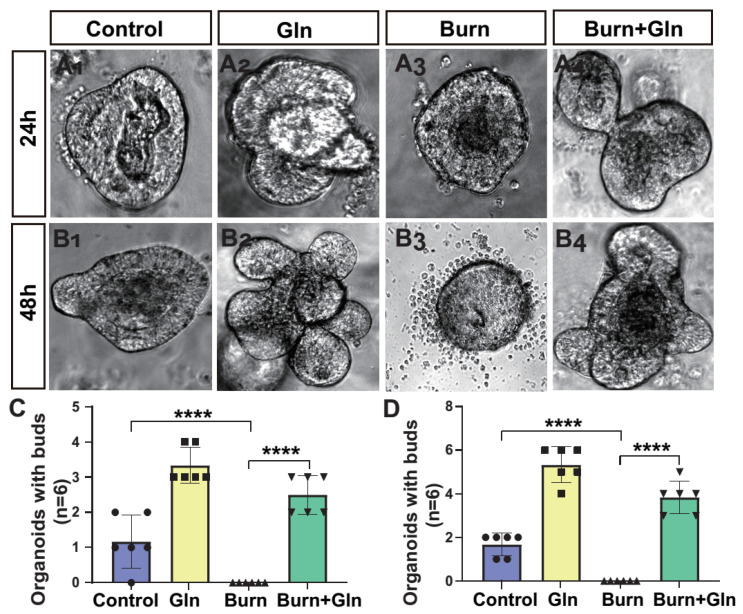
Regeneration of the organoids of the mouse small intestine crypts after Gln treatment. (**A_1_**–**A_4_**) are representative images of the organoids of the mouse small intestine crypt in control group, Gln group, Burn group and Burn+Gln group 24 h after burns injury. (**B_1_**–**B_4_**) are representative images of the organoids of the mouse small intestine crypt in control group, Gln group, Burn group, and Burn+Gln group 48 h after burns injury. (**C**) Comparison of the number of budding crypts in a single organoid of the mouse small intestine crypt 24 h after burns injury. (**D**) Comparison of the number of budding crypts in a single organoid of mice small intestine crypt post burns 48 h. Data were expressed as mean ± SEM from 6 independent experiments (*n* = 6). **** *p* < 0.0001 using 1-way ANOVA and post hoc Tukey’s test.

Subsequently, we further utilized an intestinal organoids model to determine the effects of Gln on the survival and proliferation of intestinal stem cells. It showed that the intestinal crypt organs of the control group were spherical, the hidden nest organs began to sprout in 24 h after burns, and the germination of 48 h saw a protruding germination phenotype, which is called budding in biology (Figure 2A–C). The spheroid area of organoids increased significantly in an incubation time-dependent manner. It was found that the spheres of the intestinal 3D organoid were significantly contracted and accompanied by large numbers of dead cell fragments after burns, and the buddings were reduced compared with the control group, then accompanied by large numbers of dead cell fragments. However, Gln treatment increased the number of spheres of the intestinal organs and the area of spheres (Figure 2C,D). It indicated that ISCs were seriously damaged by burn injury from the perspective of 3D organoids. In contrast, Gln treatment can relieve the damage of intestinal organoids and improve the budding rate, thereby accelerating the proliferation of intestinal organoids.

### 2.3. Effects of Gln on Endogenous Apoptosis and Ferroptosis of ISCs after Burns

**Figure 3:**
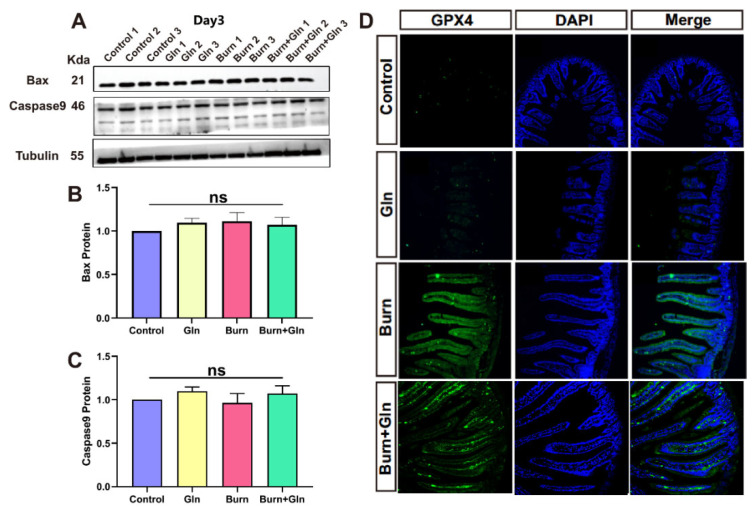
Effects of Gln treatment on apoptosis and ferroptosis in the small intestine after severe burns. (**A**) Gln supplementation after severe burns for 3 days. The expressions of Bax and Caspase9 were detected by immunoblotting (*n* = 6). (**B**) The relative intensities of Bax on the 3rd day (Day 3) after burn of bands were quantified using ImageJ. (**C**) The relative intensities of Caspase9 on the 3rd day (Day 3) after burn of bands were quantified using ImageJ. (**D**) Representative images of immunofluorescence for Glutathione Peroxidase 4 (GPX4) (green) and diamidino-phenyl-indole (DAPI) (blue): Control group; Burn group; Gln group; and Burn+Gln group. Data were expressed as mean ± SEM from 6 independent experiments (*n* = 6).

To investigate the salvage effects of Gln treatment on ISCs, we first detected the endogenous apoptosis of ISCs after burns. We verified the expression of two apoptotic proteins (BAX and Caspase9), the endogenous pro-apoptotic effectors. Due to the severe intestinal injury observed by previous H&E staining on the third day after burns, we tested the protein levels of BAX and Caspase9 in ISCs on the third day after burns. As indicated in Figure 3A,B, there were no significant differences in BAX protein levels at different time points after burns, and Gln supplementation and the repeats of each group maintained consistent homogeneity. Similarly, the protein level of Caspase9 had no significant difference between each group on the third day after burns in the expression level in the control group, the Gln group, the Burn group, and the post-burn treated with the Gln group (Figure 3C). Subsequently, we verified the changes of ferroptosis marker GPX4 in ISCs by immunofluorescence on the third day after burn injury. A large number of GPX4^+^ cells were found in the Burn group compared with the control one, and we speculated that the increase in GPX4 in the Burn group may be due to the stress states of the body after burns. However, the fluorescence of GPX4+ cells decreased after Gln treatment on ISCs after burns, which indicated that the addition of Gln could decrease the ferroptosis of crypt cells in the ileum. This may be related to the antioxidant properties of Gln itself (Figure 3D). These results suggested that the damages of ISCs after burns were unrelated to endogenous apoptosis. However, the addition of Gln alleviated ferroptosis of ISCs after burn injury.

### 2.4. Gln Treatment Promotes Self-Renewal of ISCs by Speeding Up the Cell Cycle and Promoting Proliferation

**Figure 4:**
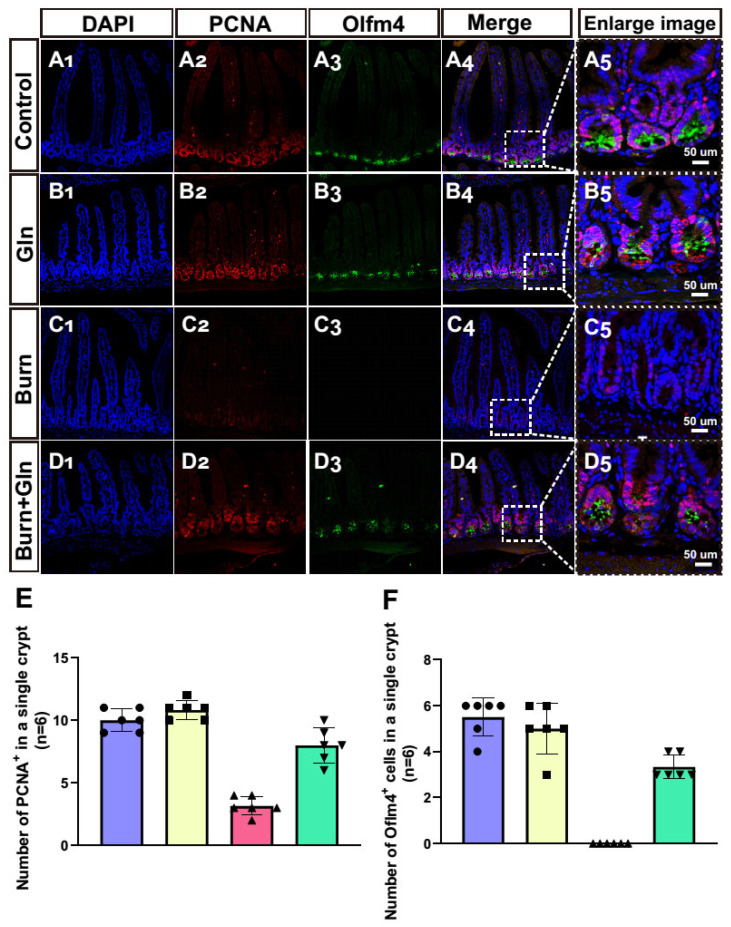
Influences of Gln treatment on proliferation and stemness of small intestinal crypts and ISCs of C57 mice after burn injury. (**A**–**C**) Representative images of immunofluorescence for proliferation cell nuclear antigen (PCNA) (red), olfactomedin 4 (Olfm4) (green), and DAPI (blue) in different groups: (**A_1_**–**A_5_**) Control group. (**B_1_**–**B_5_**) Gln group. (**C_1_**–**C_5_**) Burn group. (**D_1_**–**D_5_**) Burn+Gln group. (**E**) Comparison of the number of PCNA-positive cells in a single crypt in different groups. (**F**) Comparison of the number of Olfm4-positive cells in a single crypt in different groups. Data were expressed as mean ± SEM from 6 independent experiments (*n* = 6).

**Figure 5:**
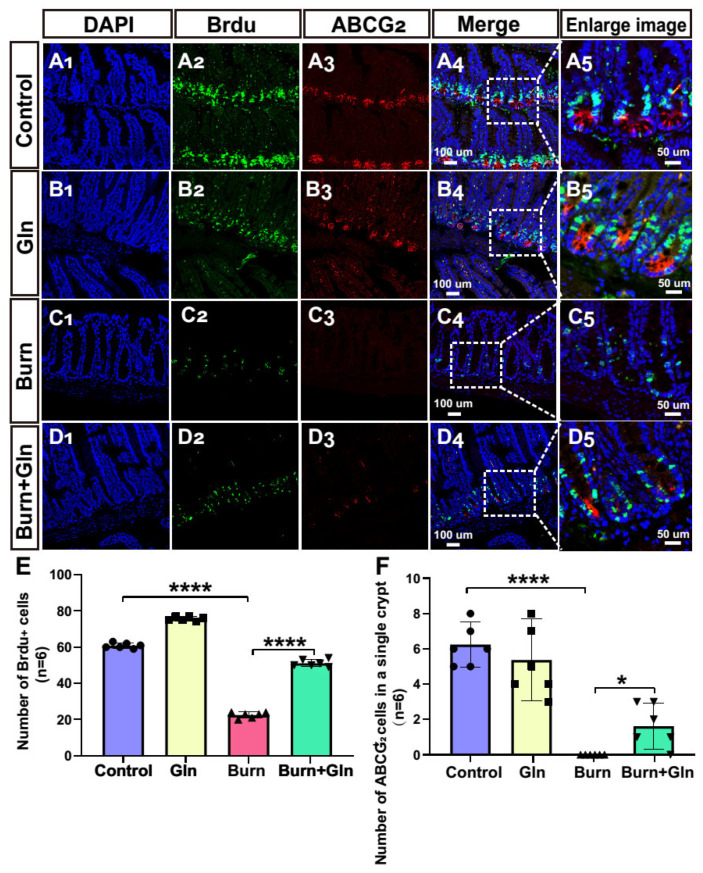
Gln treatment accelerates the small intestinal stem cell cycle and promotes the dry expression of ISCs C57 mice after burn injury. (**A**–**D**) Representative images of immunofluorescence for BrdU (**green**), ATP binding cassette subfamily G member 2 (ABCG_2_) (red), and DAPI (blue) in different groups: (**A_1_**–**A_5_**) Control group. (**B_1_**–**B_5_**) Gln group. (**C_1_**–**C_5_**) burn group. (**D_1_**–**D_5_**) Burn+Gln group. (**E**) Comparison of the number of BrdU-positive cells in a single crypt in different groups (*n* = 6). (**F**) Comparison of the number of ABCG_2_-positive cells in a single crypt in different groups. Data were expressed as mean ± SEM from 6 independent experiments (*n* = 6). * *p* < 0.1, **** *p* < 0.0001 using 1-way ANOVA and post hoc Tukey’s test.

**Figure 6:**
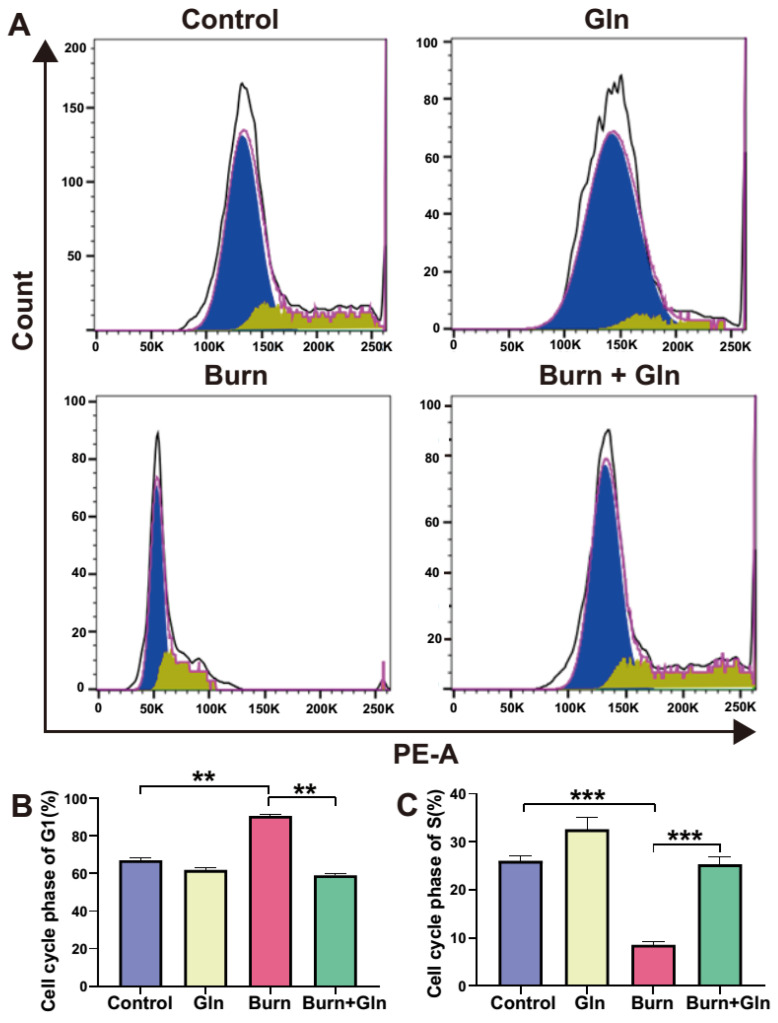
Gln could promote proliferation and accelerate cell cycle. (**A**) Flow cytometry was used to analyze the typical cell cycle of small intestinal stem cells treated in different groups. (**B**) The proportion of G1 phase of cell cycle in different groups. (**C**) The proportion of S phase of cell cycle in different groups. Data were expressed as mean ± SEM from 6 independent experiments (*n* = 6). PE-A represents the curve area of fluorescence intensity. The blue part represents the cell cycle phase of G1 and the yellow part represents the cell cycle phase of S. ** *p* < 0.01, *** *p* < 0.001 using 1-way ANOVA and post hoc Tukey’s test.

Apart from the endogenous apoptosis and ferroptosis effect, we next measured the influence of Gln on the cell proliferation and cell stemness of ISCs. Firstly, we assessed the proliferation of each group after burn injury. Crypts were marked by the white boxes on each image (Figure 4A–D), and the corresponding magnified images were displayed in Figure 4(A_5_–D_5_). The number of proliferation cell nuclear antigen (PCNA)-positive cells was greatly decreased after burns, and Gln supplementation reversed the burn-induced inhibitory effects on ISCs (Figure 4A–E), indicating that Gln can maintain cell proliferation and inhibit crypt injury after burns. Olfm4 is a stem cell marker of ISCs. Similarly, the number of olfactomedin 4^+^ (Olfm4^+^) positive cells was significantly decreased after burns, while Gln treatment significantly aggrandized the number of Olfm4^+^ positive cells compared with the Burn group, especially at crypt bottoms, which was the location of ISCs (Figure 4A–D,F). Furthermore, we used 5-bromodeoxyuridine (BrdU) staining to identify and quantify cells undergoing mitosis. We found that the BrdU-positive cells in the crypts were significantly reduced after burns, indicated that the cell cycle delay in ISCs. However, the expression of BrdU-positive cells was significantly elevated in the Burn+Gln group (Figure 5A–E). In addition, the trends of ATP binding cassette subfamily G member 2 (ABCG_2_), another stemness marker, were consistent with the trends of Olfm4 results (Figure 5A–D,F). To further determine the regulation of the cell cycle in ISCs by Gln supplementation after burn injury, we examined the cell cycle by flow cytometry. The results showed that the proportion of the G0/G1 phase was significantly increased, and the proportion of the S phase was decreased after burns. However, Gln supplementation reversed the cell cycle changes after burns and promoted the transition of the G1-S phase in ISCs (Figure 6A–C). These results indicated that complementary Gln treatment accelerates the cell cycle and promotes ISCs proliferation after burns in vitro, which was consistent with the results of PCNA staining.

**Figure 7:**
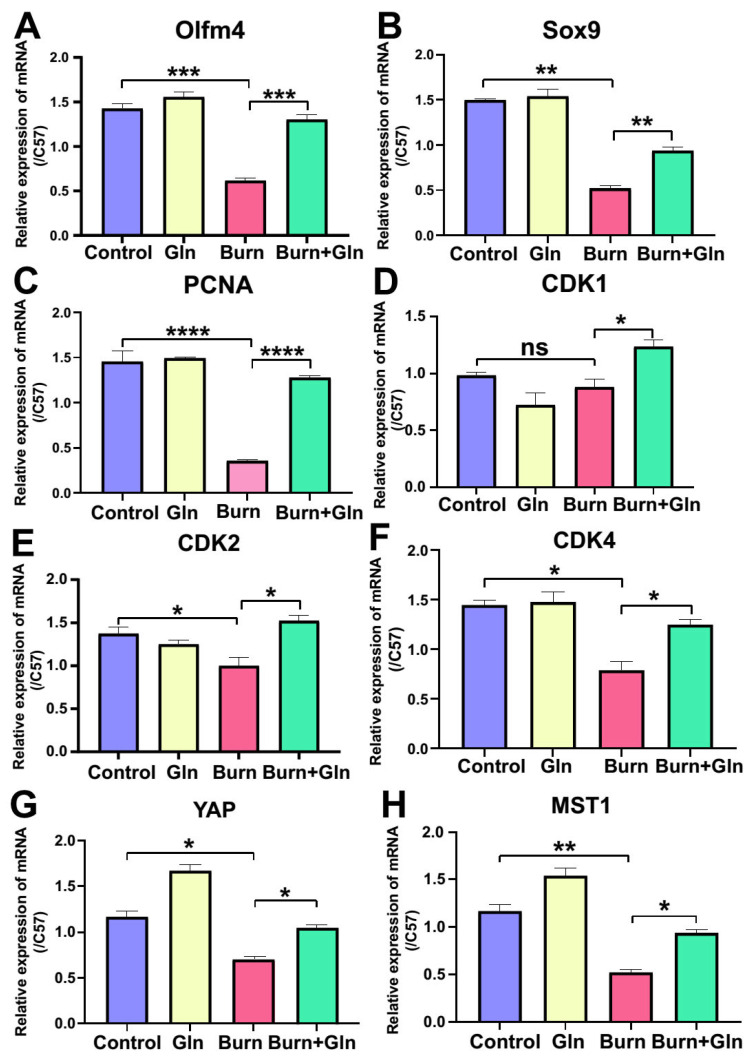
Gln accelerates the small intestine stem cell cycle process and promotes self-renewal. (**A**–**C**) The expression of the mRNA with stem cell markers in the four different groups according to the quantitative by Real-Time Quantitativeq Polymerase Chain Reaction (RT-qPCR). (**A**) Olfactomedin 4 (Olfm4) mRNA expression. (**B**) Sulfur Oxides 9 (SOX9) mRNA expression. (**C**) Proliferation Cell Nuclear Antigen (PCNA) mRNA expression. (**D**–**F**) The expression of the mRNA related to cell cycle of mouse small intestine crypts was measured by RT-PCR from the 4 different groups on day 3 after burn: (**D**) Cyclin-Dependent Kinase12 (CDK1) mRNA expression. (**E**) Cyclin-Dependent Kinase 2 (CDK2) mRNA expression. (**F**) Cyclin-Dependent Kinase 4 (CDK4) mRNA expression. (**G**) Yes-associated Protein (YAP) mRNA expression. (**H**) Mammalian sterile 20-like kinase 1 (MST1) mRNA expression. Data were expressed as mean ± SEM from 6 independent experiments (*n* = 6), ns no significance, * *p* < 0.1, ** *p* < 0.01 *** *p* < 0.001, **** *p* < 0.0001 using 1-way ANOVA and post hoc Tukey’s test.

To further clarify the regulatory effects of Gln on the proliferation and stemness of ISCs after burns, the mRNA levels of the targets that related to proliferation and stemness were validated by Real-Time Quantitativeq Polymerase Chain Reaction (RT-qPCR). The results showed that the mRNA levels of Sulfur Oxides 9 (SOX9), PCNA, Cyclin-Dependent Kinase 2 (CDK2), and Cyclin-Dependent Kinase 4 (CDK4) were down-regulated after burn injury but elevated by Gln supplementation. Although the levels of CDK4 were of no significance between the Burns and Control group, it up-regulated by Gln compared with the Burns group (Figure 7A–F). Hippo-Yes-associated Protein (Hippo-YAP) signaling notoriously plays a vital role in stem cell proliferation, cell cycle, and tissue regeneration in mammalians. Coincidentally, these targets related to proliferation and stemness were the signature of YAP. Additionally, the mRNA levels of YAP and MST1, which was the pivot of Hippo-YAP signaling, were remarkably decreased after burn injury, while aggrandized by Gln (Figure 7G,H). These results revealed that the proliferation and stemness of ISCs were eroded after burns, and Gln supplementation could antagonize the damnification, thereby promoting the endogenous proliferation of ISCs to maintain the renewal of intestinal epithelial cells.

### 2.5. Gln Promotes Proliferation, Stemness and Modulates Cell Cycle Progression of ISCs via an Activation of YAP

**Figure 8:**
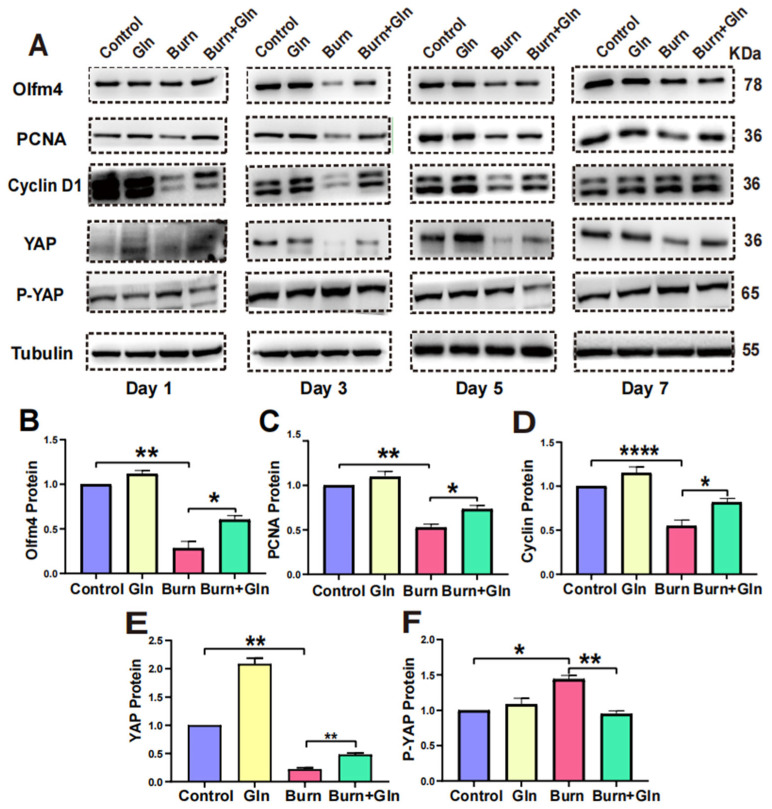
Gln promotes proliferation by activating the YAP-Hippo signaling pathway. (**A**) Protein abundances in crypts from C57 mice of Olfm4, PCNA, Cyclin D1, YAP, and p-Yes-associated Protein (p-YAP) were measured by immunoblotting using antibodies in small intestinal stem cell tissue treated with Gln after burn (*n* = 6). (**B**) Quantification of Olfm4 protein expression in crypts from C57 mice on Day 3. (**C**) Representative immunoblot analysis of PCNA in treated Gln. (**D**) Quantification of Cyclin D1 protein expression in crypts from C57 mice on Day 3. (**E**) Quantification of YAP expression in crypts from C57 mice on Day 3. (**F**) Quantification of p-YAP expression in crypts from C57 mice on Day3. Data were expressed as Mean ± SEM from 6 independent experiments (*n* = 6). * *p* < 0.1, ** *p* < 0.01, **** *p* < 0.0001 using 1-way ANOVA and post hoc Tukey’s test.

To determine whether Gln promotes proliferation and stemness and modulates the cell cycle of ISCs after burn injury through activating YAP. We detected the protein levels of YAP and p-YAP by Western blot. As shown in Figure 8A, the levels of Olfm4, PCNA, Cyclin D1, and YAP were significantly decreased in the Burn group compared with control one on the first and third day, whereas they were remarkably elevated in the Burn+Gln group. Together, the level of p-YAP was ascending by burn injury on the first and third day while declining in the Burn+Gln group. In addition, the changes of these proteins on the fifth and seventh days were slight compared to the first and third days in the Burn group, but there was still a facilitative effect of Gln on these proteins (Figure 8A–F). These results suggested that YAP was restrained after burn injury, and Gln supplementation could increase the activation of YAP.

## 3. Discussion

Severe burns can be led to intestinal barrier damage and inhibit its repair, which in turn triggers a series of pathophysiological changes. Determining methods to mitigate burn-induced intestinal injury and promote repair is one of the core issues in burn surgery and intensive care medicine. In this research, we demonstrated that the intestinal pathological tissues and crypts were seriously damaged after burns. Gln treatment alleviated the damage to the pathological structure and crypts of the small intestine. Then, the experimental results certified that Gln supplementation facilitated the proliferation repair and self-renewal of ISCs by accelerating the cell cycle, thereby alleviating the injury of ISCs. Finally, this study found that the mechanism of Gln promoted the proliferation and the repair of damaged intestinal mucosa of ISCs after burn injury by activating YAP.

A previous study showed that Gln has beneficial effects on the repair of intestinal barrier damage post-burn by providing fuel for metabolism and alleviating intestinal injury, accelerating intestinal repair [9]. Whereas the impact of Gln treatment on ISCs has been reported only in very few studies [20]. Gln has an antioxidant function, which plays an important role in maintaining the redox balance of the body [8,21]. This study verified that Gln treatment decreased small intestinal injury scores, mitigated pathological injury, increased the length of the crypt, relieved crypt atrophy, and improved the survival of the crypts of small intestinal. In addition, supplementation with Gln had no significant changes in the expression levels of Caspase9 and BAX proteins of ISCs after burns (Figure 3A–C), indicating that Gln had no effects on endogenous apoptosis of ISCs. Studies have reported that ferroptosis is a new type of programmed cell death [22]. It is iron-dependent and with characteristics mainly of oxidative stress and different from apoptosis, cell necrosis, and autophagy [23]. However, Gln supplementation could decrease the ferroptosis of crypt cells in the ileum. Therefore, we were convinced that the mechanism by which Gln could alleviate the damages of ISCs and repair intestinal mucosa was independent of endogenous apoptosis while dependent on ferroptosis.

This study focused on the effects of Gln treatment on the proliferation and self-renewal of ISCs for experiments. It is reported that regulating ISC proliferation can accelerate nutrient metabolism and promote small intestinal maturation [12]. Several studies have shown that the promotion of Gln on intestinal cell proliferation is also observed in rats [24], chicks [25], and mice [26]. Our experiments also revealed that Gln treatment increased the ISCs proliferation markers (PCNA and BrdU) and stemness markers (Olfm4 and ABCG_2_) in the crypts after burns. Interestingly, we found that the proliferating cells overlapped with ISCs in the crypts, speculating that these cells are likely to be progenitor cells [27]. The regeneration of the intestinal epithelium is driven by active ISCs at the bases of the crypts. The multipotent ISCs proliferate continuously to replace the old epithelial cells to maintain the integrity of the epithelial cells [28]. The higher quantities and percentages of multipotent cells mean an increase in the stem cells niche. A series of studies have demonstrated that cyclins play a role in enhancing pluripotency and promoting proliferation [29,30]. It was found that Gln treatment increased the ratio of cell cycle G1 of ISCs and promoted the transcription levels of cyclin CDK1/CDK2/CDK4 in our research. Cyclin D1 is a key factor in the proliferation of ISCs and is a target of the Hippo-YAP signal [31]. This study found that the supplementation of Gln promoted the expression of protein Cyclin D1 and improved ISCs proliferation after burns. Therefore, we believed that the reason why Gln supplementation promotes the proliferation, repairment, and self-renewal of stem cells after burns are due to the acceleration of the process of the stem cell cycle.

Some literature has confirmed that the alteration of YAP activity plays an important role in regulating cell injury response and post-injury repair [32]. The Hippo pathway governs organ size and tissue homeostasis in higher-order vertebrates by regulating cell proliferation and cell death [33]. YAP is a key effector in the Hippo pathway, which can promote stem cell proliferation and tissue regeneration [34]. The point of view of improving stem cell proliferation and self-renewal and promoting the phosphorylation of YAP could cause epithelial regeneration in ulcerative colitis post-inflammatory [35,36]. In order to deepen the mechanism, we clarified the regulatory relationship between MST1 in regulating the proliferation and development of ISCs through the Hippo-YAP signaling pathway. In mammals, the core of the Hippo pathway is a kinase cascade, and MST1 and YAP families are considered the key components [37,38]. In this study, it was found that Gln administration could promote the activation of YAP significantly, thereby promoting the proliferation of ISCs. Previous studies have demonstrated that the activation of YAP can promote the repair of cardiac damage [39]. Therefore, this study evidenced that the activation of YAP after Gln supplementation may explain the enhancement in the proliferation and stemness of ISCs after burns. This study demonstrated the supplementation of Gln could decrease the MST1 expression and promote the activation of YAP after burns. It was suggested that MST1 is the upstream regulatory transcription factor of the YAP protein.

In addition, the results of this paper are also verified in the literature. For instance, the activation of YAP is altered when MST1 is inactivated in response to external signals in mammals, such as low cell density, to regulate the YAP downstream domain family of transcription factors TEAD [40]. This can regulate cell proliferation and death. MST1 is homology with YAP and could act as a transcription coactivator, but there is a lack of a linear relationship between MST1 and YAP. Therefore, we speculated that the change in the activation of YAP might be regulated by other YAP regulatory factors besides MST1, such as TEAD. To further explore these issues, future studies will need to observe changes in ISCs in MST1-deficient mice. MST1 has multiple targets and may regulate YAP through multiple targets rather than one. These will become the next research focus of our future study.

## 4. Conclusions

This study demonstrated for the first time in the burned model and 3D organoid model that Gln supplementation alleviated proliferation inhibition and promoted self-renewal of ISCs to mitigate burn-induced intestine damage. Gln supplementation could promote the activation of YAP, expedite the cell cycle of ISCs, and alleviate intestinal mucosa damage post-burn. These findings deepen the understanding of the mechanism of Gln promoting the repair of the damaged intestinal mucosa and helping to find new regulatory targets to maintain the intestinal mucosal barrier after burns.

## 5. Material and Methods

### 5.1. Experimental Animals

The mice used in the experiment were all male C57BL/6, which were fed in the specific pathogen-free (SPF) grade animal facilities of the Clinical Medical Research Center, Southwest Hospital of Third Military Medical University. The mice were kept in the animal feeding room for one week to adapt to the environment, and then the burned mouse model was constructed. All animal experiments were conducted in strict accordance with the national and Third Military Medical University guidelines for the use of experimental animals and were approved by the Medical Ethics Committee of Third Military Medical University (ethical approval code: AMUWEC20224200).

### 5.2. Murine Model of Burn Injury

Based on the previous study, we constructed a burned mouse model [41]. Briefly, the 1% pentobarbital sodium (40 mg/kg) was used to anesthetize mice; the hair on the back of mice was removed with a razor and coated with hair removal cream for 20 s. Additionally, the cream was gently wiped away with medical gauze soaked with PBS so that the exposed area was about 30% of the total surface of the body. According to the experimental requirements, the mice were randomly divided into 4 groups, named Control group, Gln group, Burn group, and Burn+Gln group. The hair-free backs of mice were fixed with a special hollowed-out container so that the backs leaked out at the bottom. The backs of mice were placed in a 95 °C water bath for 12 s, then the water on the backs of mice was dried, and 1 mL of normal saline was injected into each mouse for rehydration. Gln treatment was used with intraperitoneal injection after the mice woke up, and the same volume of saline was injected in Control group. The burned mice were kept at 37 °C for 72 h and divided into 1 cage per mouse to ensure that the burned area would not be scratched by other mice.

### 5.3. H&E Staining and Determination of Villi Length and Crypt Depth

As described in previous study [21], we made tissue sections using small intestine tissue and performed H&E staining. Small intestine tissues of mice at different time points were taken, and the ileum was fixed with 4% paraformaldehyde. The small intestine was paraffin-embedded and sliced into 5 μm sections for long-term preservation and follow-up experiments. The paraffin sections were dewaxed and stained with hematoxylin and eosin (H&E). The staining effect was observed under microscope, then taken photos to evaluate the morphological changes of intestinal villi and crypt in each group. The Fiji ImageJ 1.8.0.345 software was used to measure the villi length and crypt depth.

### 5.4. PAS Staining

Goblet cells were stained using the periodic acid Schiff staining kit (Beyotime, Shanghai, China) [42]. Briefly, the paraffin sections of each group were first dewaxed with xylene and gradient ethanol, cleaned with distilled water, purified with iodate for 10 min, rinsed with tap water for 10 min and Schiff solution for 10 min, rinsed with PBS for 5 min, and nucleated with harisoxylin or Meyer hematoxylin for 3 min (too deep nuclear staining can be differentiated by hydrochloric acid alcohol). They were rinsed with running water for 5 min. Finally, routine dehydrated by gradient ethanol and xylene, sealed with neutral resin, microscopic observation, and photography records. A total of 30 experimental animals C57BL/6 mice were used.

### 5.5. Crypt Isolation and Culture

As described in previous study [43], crypt isolation: Fresh intestine tissues were taken from mice and rinsed with normal saline to remove contaminants, the intestine was cut lengthwise, and the inner wall of the intestine was gently rinsed. Additionally, they were then cut into small sections of 0.5 cm, impregnated in a centrifuge tube containing 40 mL sterile chelating agent (1X dissociation buffer, Ethylene Diamine Tetra-acetic Acid (EDTA) and Ethylene Glycol Tetra-acetic Acid (EGTA) 400 μL each). The tube was put in a shaker at 4 °C for 30 min, the chelating agent was discarded, and the small intestinal fragment was washed with sterile Phosphate Buffered Saline (PBS). The centrifuge tube was driven up and down with arm to separate the crypts, and the 70 μm filter was used to separate the villi and crypt. The filtrate was centrifuged at 700 rpm for 10 min, and the white precipitate on the bottom of tube was intestinal crypts. The obtained crypts were divided into three parts for culture, one part was used for culture, one part was used for extraction of total protein, and the other part was used for extraction of total RNA.

Crypt organoid culture: The obtained crypts were added to 40 mL sterile PBS containing BSA for re-suspension and centrifuged at 700 rpm at 4 °C for 5 min, and supernatant was removed. 0.5 mL washing medium was added (10 mL was prepared according to the ratio of 9.7 mL F12 base medium (Gibco, San Diego, CA, USA), 100 μL Penicillin Streptomycin (Beyotime, Shanghai, China), 100 μL Glutamax (Gibco, San Diego, CA, USA), and 100 μL NAC (Sigma, Reeds Spring, MO, USA)) to resuspend the crypts. The crypts were settled naturally for 5 min (this step can be omitted if a small number of crypts are collected), the total volume of crypts can be calculated directly according to the number of crypts required by each hole (10–30 crypts/10 μL matrix glue/96 holes). The corresponding volume of recess suspension was taken and centrifuged at 700 rpm at 4 °C for 5 min. The supernitant was discarded, and the corresponding volume of matrigel was added to the ice to re-suspend the crypts. The matrigel was dispensed at 10 μL per well to the center of the 96-well plate and then promptly placed in the incubator at 37 °C for 17 min. The 10 mL ISC culture medium consisted of 9.5 mL DMEM (Gibco, San Diego, CA, USA), 200 μL B27 (Gibco, San Diego, CA, USA), 100 μL N2 (Gibco, San Diego, CA, USA), 100 μL Glutamax (Gibco, San Diego, CA, USA), 100 μL Penicillin Streptomycin (Beyotime, Shanghai, China), and 10 μL NAC (Sigma, Reeds Spring, MO, USA). 100 μL complete medium (7 mL IntestiCult™ OGM (Stemcell, Vancouver, BC, Canada) and 100 μL Y27632 (Sigma, Reeds Spring, MO, USA), 3 mL ISC culture medium) were added to each well, and the medium was changed every 2 days. A total of 30 experimental animals C57BL/6 mice were used.

### 5.6. Protein Extraction and Western Blot Analysis

Based on the previous study [21], 200 μL Radioimmunoprecipitation (RIPA) solution (Beyotime, Shanghai, China), which contains 1x protease inhibitor, was added to crack the crypts and shaken at 4 °C for 20 min to fully crack the crypts. The protein concentration was determined by Bicinchoninic Acid (BCA) kit (Beyotime, Shanghai, China). After adding the protein loading buffer, the protein was denatured at 100 °C for 10 min and stored at −80 °C refrigerator. The protein samples with the calculated concentration were subjected to Sodium Dodecyl Sulfate Polyacrylamide Gel Electrophoresis (SDS-PAGE) gel electrophoresis. Then, they were gummed, transferred, sealed, and incubated primary antibody overnight at 4 °C. Protein primary antibody information and dilution ratio: BAX (1:1000, Proteintech, Wuhan, China), Caspase9 (1:1000, CST, Boston, MA, USA), PCNA (1:1000, Abcam, Cambridge, UK), Olfm4 (1:1000, CST, Boston, MA, USA), P-YAP (1:1000, Abclonal, Wuhan, China), YAP (1:1000, Abcam, Cambridge, UK), Cyclin D1 (1:1000, CST, Boston, MA, USA). Finally, we incubated secondary antibodies, and the ECL solution was used to reveal the bands. A total of 30 experimental animals C57BL/6 mice were used.

### 5.7. Immunofluorescence

As described in previous study [21], paraffin sections of mouse small intestine were used for immunofluorescence analysis. The paraffin sections were first baked at 60 °C for 2 h, then dewaxed and cleaned with PBS. The tissue sections were placed in 0.01 M sodium citrate buffer for antigen repair by microwave method, cleaned with PBS, and then coated with 3% hydrogen peroxide solution. Incubated in a wet box for 15 min to inactivate endogenous peroxidase. Goat serum was added and incubated at room temperature for 20 min to block the nonspecific antigen. The serum was discarded, and diluted primary antibody working solution was added to incubate overnight at 4 °C. Protein primary antibody information and dilution ratio: Olfm4 (1:100, CST, Boston, MA, USA), PCNA (1:100, Abcam, Cambridge, UK), Cyclin D1 (1:100, CST, Boston, MA, USA), GPX4 (1:100, CST, Boston, MA, USA), BrdU (1:100, CST, Boston, MA, USA), Olfm4 (1:100, CST, Boston, MA, USA), Cyclin D1 (1:100, CST, Boston, MA, USA), followed by drops of Alexa Fluor 488 or 594 fluorophores (Invitrogen, CA, USA), incubated at room temperature for 30 min against light, and sealed with anti-fluorescence quenching tablets, and confocal laser imaging was used. A total of 30 experimental animals C57BL/6 mice were used.

### 5.8. Cell Cycle Detection

As described in previous study [44], the isolated crypts were transferred to a new 15 mL centrifuge tube, resuspended with 2 mL TrypLE Express (Gibco, San Diego, CA, USA) digestive solution, and incubated at 37 °C for 5–10 min. After being blown and re-suspended several times with a 1 mL pipette, 10 μL cell suspension was taken for microscopic examination. A final concentration of 10% FBS was added, and the cell suspension was filtered through 40 μm cell sieve. The suspensions of the filtered crypts were centrifuged at 4 °C for 5 min at 500× *g*. Cells were cleaned with Basal medium twice and centrifuged at 4 °C for 5 min. Precooled 70% ethanol was added, gently blown and mixed, fixed at 4 °C overnight, and centrifuged at 1000× *g* for 5 min; the supernatant was discarded; the cells were cleaned with precooled PBS; propyl iodide staining solution was added; the cells were stained at room temperature for 30 min; the flow cytometry was used to detect the changes of cell cycle. A total of 30 experimental animals C57BL/6 mice were used.

### 5.9. RNA Extraction and Quantitative Real-Time PCR Analysis

As described in previous study [45], the isolated crypts were cracked by TRizol (Takara, Osaka, Japan) on ice for 10 min, the chloroform was added into an upside-down tube and mixed, centrifuged at 4 °C at 12,000 rpm for 15 min. The upper water phase was transferred to a new tube, and the same volume of isopropyl alcohol was added and mixed, precipitated for 2 h at −20 °C, and centrifuged for 10 min at 12,000 rpm at 4 °C. The white precipitate at the bottom of the tube was RNA. The RNA was washed twice with 75% ice ethanol and dissolved in enzyme-free water after air drying in the ventilation cabinet. Nanodrop 2000 was used to measure the concentration and purity of RNA, the performed reverse transcription (Takara, Osaka, Japan), and qRT-PCR (Takara, Osaka, Japan) according to the kit instructions. A total of 30 experimental animals C57BL/6 mice were used.

### 5.10. Statistical Analysis

All the experimental data in this study were carried out in three independent experiments. Results were tabulated with mean ± standard deviation (SD). The GraphPad Prism 8.0 software was used to conduct single-sample *T*-test for the significant difference between the two groups, the difference between multiple samples was tested by Analysis of Variance (ANOVA). The *p*-value: * *p* < 0.05, ** *p* < 0.01, and *** *p* < 0.001 defined a statistically significant difference between groups.

## Data Availability

All data from this study are included in the article and Supplementary Materials. Other data could be uploaded as required.

## Supplementary Materials

The following supporting information can be downloaded at: https://www.mdpi.com/article/10.3390/nu15071766/s1. Figure S1. Establishment of burn mouse model. (A) Renderings of the scab area on the back of mice in the Burn group and the Control group. (B) Body weight statistics of Burn group and Control group for 7 consecutive days after burns. Figure S2. Effects of Gln treatment on pathological damage of the small intestine after burns injury. The black arrow represents the site of injury in the small intestine. (A_1_–A_4_) Representative full-transverse sections of the proximal jejunum from control and Gln-treated mice stained with PAS staining: (A_1_) Control group; (A_2_) Burn group; (A_3_) Gln group; (A_4_) Burn+Gln group. (B_1_–B_4_) Enlarged image at the black box in (B_1_–B_4_) (*n* = 6). Table S1. List of primer.
