# Supplementary material for: Potential Effect of Glutamine in the Improvement of Intestinal Stem Cell Proliferation and the Alleviation of Burn-Induced Intestinal Injury via Activating YAP: A Preliminary Study
Source: Nutrients. 2023 Apr 4;15(7):1766. doi: 10.3390/nu15071766 (PMC10097377; doi:10.3390/nu15071766)
Supplement: Supplementary file 1 [file nutrients-15-01766-s001.zip › nutrients-2298693-supplementary.pdf]

Supplementary Material

Xia Chen [1,†], Panyang Zhang [1,†], Yajuan Zhang [1], Shijun Fan [1], Yan Wei [1], Zhifan Yang [2], Fengchao Wang [2,*] and Xi Peng [1,3,*]

1 Clinical Medical Research Center, Southwest Hospital, Third Military Medical University (Army Medical University), Chongqing 400038, China; chenxia05201995@163.com (X.C.); zhangpanyang1@126.com (P.Z.); zyj18696625642@126.com (Y.Z.); fanshijun1211@hotmail.com (S.F.); weiyan56333849@163.com (Y.W.)

2 Institute of Combined Injury, State Key Laboratory of Trauma, Burns and Combined Injury, College of Preventive Medicine, Third Military Medical University (Army Medical University), Chongqing, 400038, China; yang1269043@163.com

3 State Key Laboratory of Trauma, Burns and Combined Injury, Third Military Medical University (Army Medical University), Chongqing 400038, China

* Correspondence: fengchao.w@foxmail.com (F.W.); pxlrmm@tmmu.edu.cn (X.P.)

† These authors contributed equally to this work.

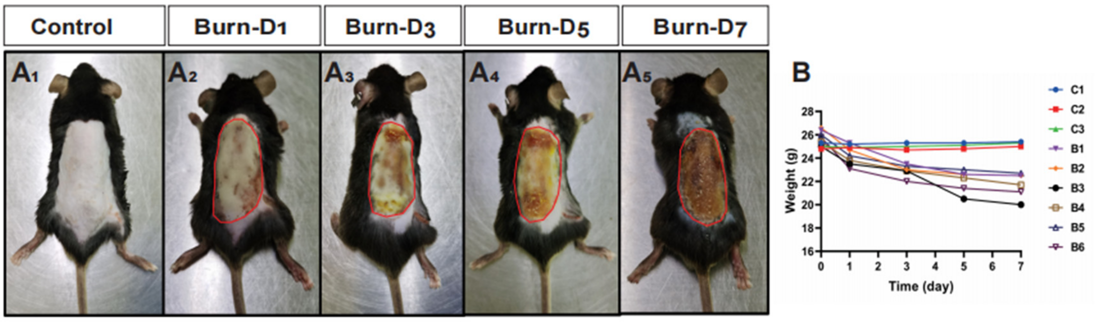

Figure S1. Establishment of burn mouse model. (A) Renderings of the scab area on the back of mice in the Burn group and the Control group. (B) Body weight statistics of Burn group and Control group for 7 consecutive days after burns.

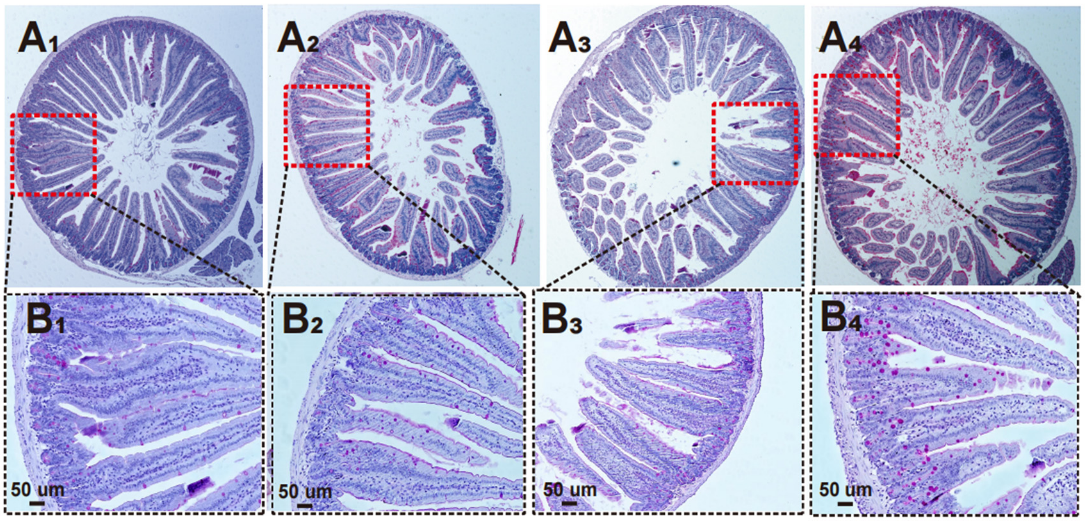

Figure S2. Effects of Gln treatment on pathological damage of the small intestine after burns injury. The black arrow represents the site of injury in the small intestine. (A_1–A_4) Representative full-transverse sections of the proximal jejunum from control and Gln-treated mice stained with PAS staining: (A_1) Control group; (A_2) Burn group; (A_3) Gln group; (A_4) Burn+Gln group. (B_1-B_4) Enlarged image at the black box in B_1-B_4 (n=6).

Table S1. List of primer

Name	Forward primer	Reverse primer
**MSTI**	CTTCCACTACAACATGAGCAGC	TGCAGGTCCGCACATAATCTT
**Yap**	TGAGATCCCTGATGATGTACCAC	TGAGATCCCTGATGATGTACCAC
**Cdk4**	CAGTCTCAGTGTCGAGCCG	TGCTCCTCCATTAGGAACTCTC
**Olfm4**	CAGCCACTTTCCAATTTCACTG	GCTGGACATACTCCTTCACCTTA
**cdk2**	GCTGGACATACTCCTTCACCTTA	CAGTCTCAGTGTCGAGCCG
**Sox9**	CAGCCCCTTCAACCTTCCTC	TGATGGTCAGCGTAGTCGTATT
**cdk1**	AGGTACTTACGGTGTGGTGTAT	CTCGCTTTCAAGTCTGATCTTCT
**PCNA**	TTGCACGTATATGCCGAGACC	GGTGAACAGGCTCATTCATCTCT
